# 

**Published:** 1854-10

**Authors:** 


Our next number will contain a large amount of original matter.

A paper on Congestion of the Brain following Cholera, by Dr. James M. Newman, will be published at the request of the City Medical Association, to whom it was read at the last meeting. It possesses unusual originality and interest.

Communications from Drs. Avery and Jameson, and Dr. N. L. North, are received, and will also appear. Their authors will please accept our thanks.

